# Hand tumors

**DOI:** 10.1590/1806-9282.2024S108

**Published:** 2024-06-07

**Authors:** Antonio Tufi Neder, Antonio Carlos da Costa, Rui Sérgio Monteiro de Barros, Luis Renato Nakachima, Mauricio Pinto Rodrigues, Sandro Castro Adeodato de Souza, Ricardo Kaempf de Oliveira, Sérgio Augusto Machado da Gama, Rodrigo Guerra Sabongi, Celso Kiyoshi Hirakawa

**Affiliations:** 1 Universidade de São Paulo/Faculty of Medicine of Ribeirão Preto - Hand Surgery at Rede Mater Dei de Saúde and Instituto Orizonti – Belo Horizonte (MG), Brazil.; 2 Medical School of Santa Casa de São Paulo, Hand Surgery and Microsurgery Group at Santa Casa de São Paulo – Belo Horizonte (MG), Brazil.; 3 Universidade do Estado do Pará, Hospital Mater Dei-Porto Dias – Belo Horizonte (MG), Brazil.; 4 Universidade Federal de São Paulo, Paulista School of Medicine, Department of Orthopedics and Traumatology – Belo Horizonte (MG), Brazil.; 5 Universidade de São Paulo, Institute of Orthopedics and Traumatology, Clinical Hospital, Faculty of Medicine – Belo Horizonte (MG), Brazil.; 6 Universidade Federal do Rio de Janeiro – Belo Horizonte (MG), Brazil.; 7 Santa Casa de Porto Alegre – Belo Horizonte (MG), Brazil.; 8 Universidade de São Paulo, Clinical Hospital, Faculty of Medicine, Pontifícia Universidade Católica de Campinas Hand Group – Belo Horizonte (MG), Brazil.; 9 Universidade Federal de São Paulo, Paulista School of Medicine – Belo Horizonte (MG), Brazil.

## SOFT TISSUE TUMORS

### Synovial cyst on the wrist

Synovial cyst (SC) is the most common soft tissue tumor in the wrist and hand, accounting for almost 50–70% of the total. It is more frequent in women, between the second and fourth decades of life. Clinically, it presents as a nodular and superficial increase in volume, which is mostly located in the dorsal and central region of the wrist (up to 80%) and originates from the radiocarpal joint (RCJ). It can also be located in the volar and radial region, which is the second most common location, or originate from the mid-carpal joint (MCJ), mainly in the scaphotrapeziotrapezoid (STT) joint, when they are located more distally^1^.

Treatment is predominantly non-surgical. However, patients with symptomatic lesions after treatment or with aesthetic complaints are candidates for invasive procedures. Aspiration, with or without corticosteroid infiltration, has a high recurrence rate, which can reach 80%, and is being less used. The surgical treatment principle consists of decompression and drainage, with resection of a portion of the joint capsule and the cyst wall, with no need for complete and extensive resection of the tumor, and with an average recurrence rate of 15%^2^.

Wrist SCs can be treated through open surgery. However, large incisions present a greater risk of general complications, such as cosmetically unsatisfactory scarring and stiffness of the wrist, and especially in the case of volar cysts, injury to noble structures such as the superficial palmar branch of the radial artery, flexor tendons, superficial terminal branches and palmar cutaneous branch of the median nerve^2,3^.

**Figure 1:**
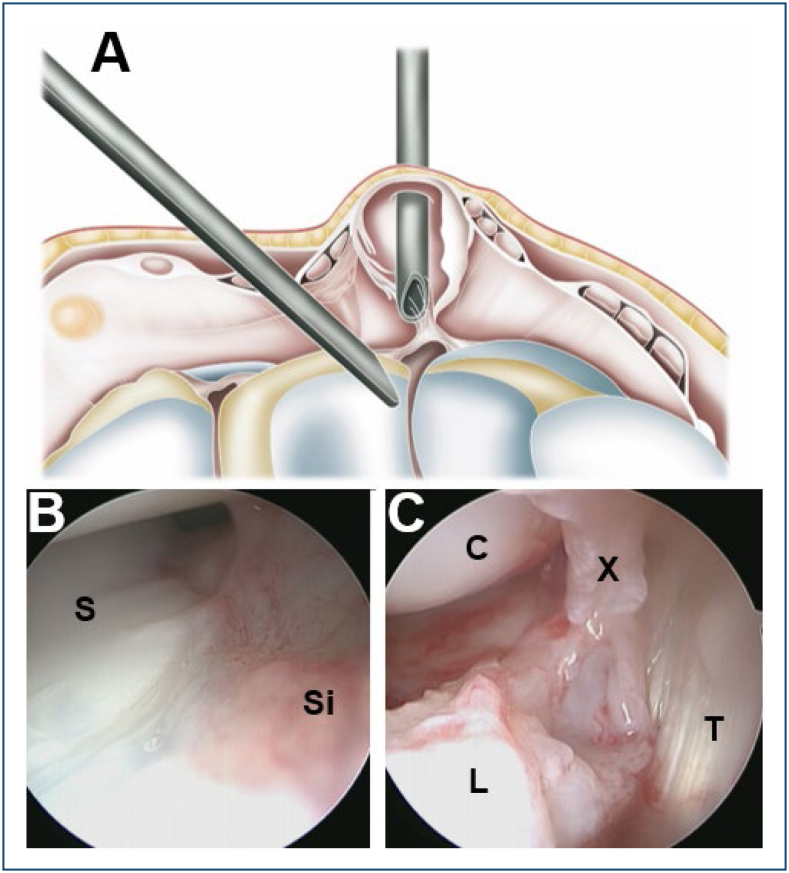
Schematic drawing showing arthroscopic surgical treatment, with the camera positioned in the mid-carpal joint, in its ulnar portal, and the shaver in the radial portal, located transcystic **(A)**. In an initial view, thickening of the dorsal capsule and synovial membrane is observed, typical of a patient with a symptomatic dorsal cyst **(B)**. Similar positioning of the optic after resection of the cyst pedicle and a portion of the dorsal capsule, making the extensor tendons visible **(C)**. S: scaphoid; Si; synovitis; C: capitate; L: lunate; X: dorsal capsule; T: extensor tendon.

Currently, arthroscopy has become a consolidated technique in the treatment of orthopedic pathologies. With technological advances, arthroscopy of small joints has allowed, through direct visualization, the diagnosis and immediate treatment of intra-articular injuries. Arthroscopic resection, initially described by Osterman^3^ for dorsal cysts and later for volar cysts, proved to be a minimally invasive alternative to the open technique (Figure 1). Its advantages are less post-operative pain, less scarring and stiffness, and a quicker return to work activities, without a high incidence of complications. The recurrence rate of wrist SCs arthroscopic treatment ranges from 0 to 26%^4-6^.

In special situations, when we do not have the appropriate material (shaver), we can use a pink needle (1.2 mm) to open the pedicle and a portion of the capsule. Due to the risk of injury to deep structures, we only recommend this "trick" to surgeons who are already trained and used to this treatment^7^.

### Giant cell tumor of the tendon sheath

Giant cell tumor of the tendon sheath (GCTTS) is one of the most common soft tissue tumors of the hand, second only to ganglion cysts. It is also known as pigmented villonodular synovitis and originates from the synovial membranes, bursae, and tendon sheaths^8^.

It affects young individuals, with women being the most affected (3:2), more commonly in the fingers. It rarely affects children^9^.

Diagnosis is mainly based on clinical examination. The GCTTS appears as a firm, painless, slow-growing mass. Ultrasound exams usually help with the diagnosis, but MRI provides more details of the tumor's characteristics.

**Figure 2:**
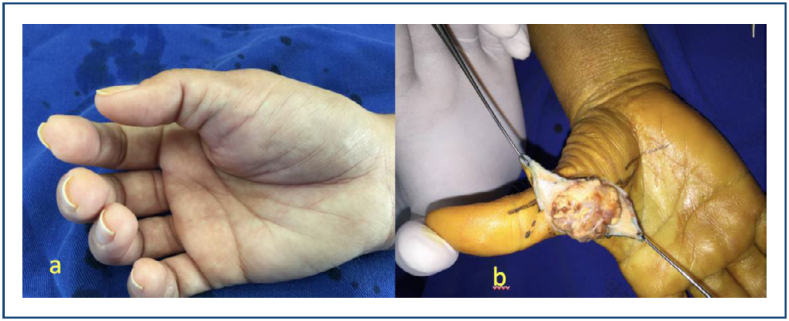
Giant cell tumor of the tendon sheath on the palmar aspect of the thumb. **(A)** Clinical appearance. **(B)** Surgical appearance of the tumor.

Diagnostic confirmation is based exclusively on anatomopathological examination. Excisional biopsy, with a safety margin, is recommended, and excision of satellite lesions is essential to avoid recurrence, which can reach up to 45% of cases. The patient should always be warned of the possibility of recurrence. Macroscopically, typical lesions are yellowish-brown and multinodular^8^ (Figure 2).

### Lipoma

Lipomas are benign tumors of fatty cells that appear as soft, painless masses. Despite being the most common tumors of mesenchymal origin, they are uncommon in the hand. They can be superficial or deep, and when located in neural pathways, they can generate compressive symptoms. The etiology of lipomas is unknown^10^.

Lipoma is a circumscribed fat nodule surrounded by a thin fibrous capsule that has a characteristic yellowish content typical of fatty tissue^11^. Although ultrasound has proven to be a useful diagnostic tool, magnetic resonance imaging is more informative, as the image shows fat signal intensity^12^.

Lipomas can be observed or excised according to their size and the patient's complaints^11^.

The definitive diagnosis is made through excisional biopsy followed by histopathological examination, which generally heals the lesion with rare recurrences.

### Glomus tumor

Glomus tumors are tumors arising from the glomus body, which is a contractile neuromyoarterial structure responsible for adjusting blood pressure and temperature and regulating blood flow to the skin^13^. Glomus tumors are uncommon and mostly benign, representing approximately 2% of all soft tissue tumors in the extremities^13^. They can be single or multiple. The most frequent clinical picture found is a female patient presenting with a small, painful, thermosensitive nodule on the nail bed^14^. Patients often report throbbing pain, "as if there was a heart under your fingernail." The tumor is often visible through the nail.

Typical symptoms of the glomus tumor triad are pinpoint pain, severe pain, and cold hypersensitivity. Diagnostic tests include the paperclip test (increased pain with localized compression), Hildreth's test (the patient's arm is elevated, a tourniquet is inflated to 250 mmHg, and the tumor is palpated. Pain and sensitivity should be reduced. The test is positive when cuff release causes sudden onset of pain and tenderness in the tumor area), and cold sensitivity test^15^.

Imaging exams also help us, which include X-rays, ultrasound, and, mainly, magnetic resonance^16^.

**Figure 3:**
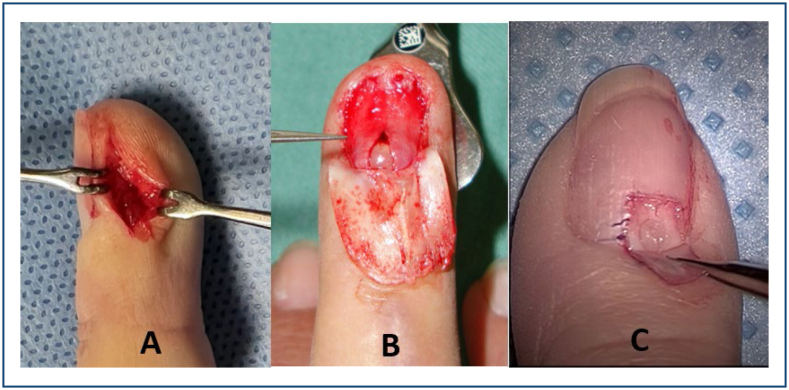
**(A)** Para-ungual access route. **(B)** Access with nail resection. **(C)** Access route through a window in the nail.

The gold standard of treatment is complete excision, which often results in permanent relief of symptoms. The tumor can be accessed through the lateral route of the finger, when the tumor is lateralized, or by resecting the nail piece. Another option is to create a "window" on the nail when the tumor is easily identified (Figure 3). Malignant presentation (glomangiosarcomas) is very rare^17^.

### Skin tumors on the hand

The hand represents only 1–2% of the body surface area, but malignant skin tumors of the hand account for about 10–15% of all malignant skin tumors. The most common malignant tumor is squamous cell carcinoma, followed by basal cell carcinoma, and finally, melanoma^18^.

Squamous cell carcinoma originates from epithelial keratinocytes and, when restricted to the epidermis, is called squamous cell carcinoma in situ or Bowen's disease. Numerous non-invasive or minimally invasive treatments are effective in this case. In invasive cases, surgical treatment is required. The American Cancer Committee recommends staging squamous cell carcinoma into low-risk tumors or high-risk tumors based on the risk of local spread or metastases. The tumors of more than 2 cm in diameter or that have at least two of the following risk factors are considered high-risk tumors: undifferentiated or poorly differentiated histology, 2 mm or more in thickness, invasion of the reticular dermis (Clark IV), or perineural invasion^19^. The study by Brodland and Zitelli^20^ suggests a 4-mm margin for low-risk tumors and a 6-mm margin for high-risk tumors. However, controversy still persists regarding the type of surgical treatment and the exact safety margin required in invasive cases.

Basal cell carcinoma is the most common malignant tumor in the human body, but it is much less common in the hand than squamous cell carcinoma. The known relationship between basal cell carcinoma and solar irradiation could contribute to a high incidence in the dorsal region of the hand, which is permanently exposed, but this does not occur, probably due to the lower proportion of sebaceous glands on the dorsum of the hand^21^. This tumor is more common in white men over 60 years of age, in immunosuppressed patients, and in patients who have already had malignant hair follicle tumors in other regions^22^.

Melanoma on the hand has a more invasive behavior than in other regions, with high rates of lymph node involvement and deaths. In terms of location, the most common is the subungual, followed by the back of the hand, and finally the palmar region. Treatment varies depending on location and depth, and wide resection and reconstruction or amputations with or without reconstructions can be performed^21^.

## BENIGN BONE TUMORS

### Enchondroma

**Figure 4:**
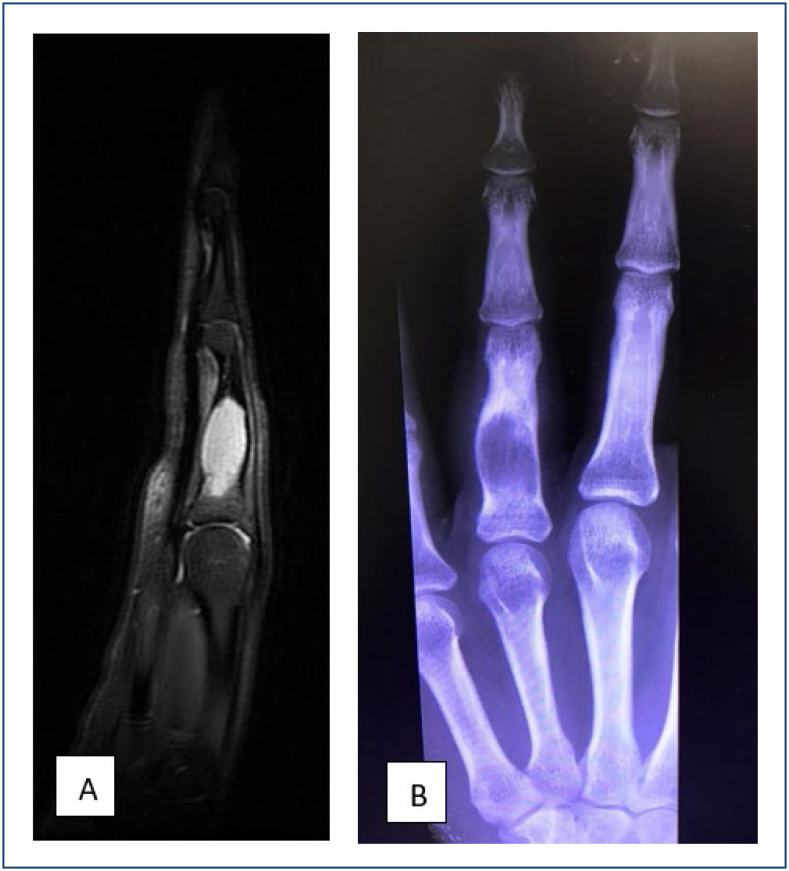
**(A)** T2-weighted magnetic resonance imaging, showing an enchondroma of the proximal phalanx of the left fourth digit, with high signal intensity of the lesion and cortical thinning. **(B)** X-ray AP view of the left hand showing a lytic, expanding lesion with cortical thinning in the proximal phalanx of the fourth finger, suggestive of enchondroma.

Enchondroma is the bone tumor that most affects the hand, accounting for 90% of cases. It is a benign lesion of the cartilaginous matrix, with a predilection for the proximal phalanges, followed by the metacarpals, and middle phalanges^23^. The initial presentation is quite variable, ranging from a finding on a radiological examination (X-ray, computed tomography (CT), or magnetic resonance imaging) carried out for another reason (Figure 4A), such as complaints of pain and increased volume at the site of the lesions, to cases of pathological fractures^24^. The radiographic characteristic is similar to that of a lytic lesion, initially involving the metaphyseal area and then expanding to include diaphyseal and epiphyseal extensions, some internal calcifications, and mild to moderate degrees of expansion and thinning of the cortical bone (Figure 4B). Treatment depends on the appearance of the lesion. Small, asymptomatic lesions "accidentally" diagnosed by tests carried out for other reasons can be monitored only clinically. In symptomatic cases, those accidentally diagnosed with dimensions in which the risk of pathological fracture is high, or even in cases of pathological fracture, surgical treatment is recommended^23^. In the specific case of pathological fractures, there is some evidence that tumor treatment has better results after fracture consolidation. Considering the very characteristic appearance of the lesion, its high frequency in the hand region, and the very low degree of malignant transformation (<5%)^25^, biopsy is generally unnecessary. Resection is performed by creating a bone window and intralesional curettage. There is no need for an adjuvant method. Filling the cavity with autologous bone graft is the most used method, but the use of heterologous grafts, synthetic substitutes, or even no filling^26^ is reported. The expected recurrence rate is low (2–15%)^25^.

### Osteochondroma

**Figure 5:**
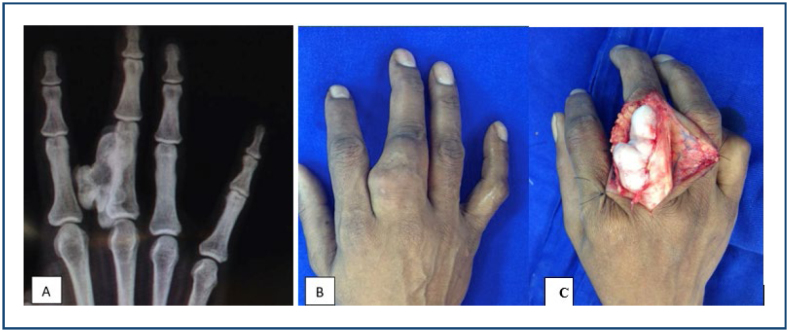
**(A)** X-ray AP view showing massive osteochondroma on the proximal phalanx of the third finger of the right hand. **(B)** Clinical image. **(C)** Exposure of the osteochondroma, with the hyaline cartilage layer being observed.

Although osteochondroma is relatively uncommon in its isolated form, it is one of the most common bone lesions of the skeleton and is more frequently found in the form of hereditary multiple osteochondromatosis. It generally appears between the second and third decades of life. According to histopathology, it is a bone growth with a layer of hyaline cartilage (Figure 5), generally originating from a herniation of the growth plate through the periosteum or the tendon insertion region and maintaining contact with the original innermost part of the bone. In the hand, the most common location is on the back of the proximal phalanges. Treatment for asymptomatic cases is observation only. For cases of angular or rotational deformity, pain, limitation of movement due to mechanical blockage, nerve compressions, prominence, irritation, and even tendon rupture, surgical treatment is recommended^23^. Malignant transformation in isolated chondrosarcomas is generally low (1%) and not reported in specific cases of the hand. In cases of multiple osteochondromatosis, it can be 2–5%^24^.

### Osteoid osteoma

Skeletal bone tumors can be divided into benign, primary malignant, and metastatic. Osteoid osteoma (OO) is a benign neoplasm that rarely occurs in the hand bones and is often difficult to diagnose. It mainly affects males (61.5%), aged between 20 and 29 years (53%), and is most frequently found in the phalanges (52.9%), mainly in the proximal phalanx, followed by the metacarpals (14.5%) and distal phalanx (13%). The occurrence in the carpal bones is lower, with the scaphoid being the most affected (7.7%). The most frequently observed location is within the cortical bone (intracortical), followed by cancellous bone, subperiosteal, and juxta-articular regions^27^.

**Figure 6:**
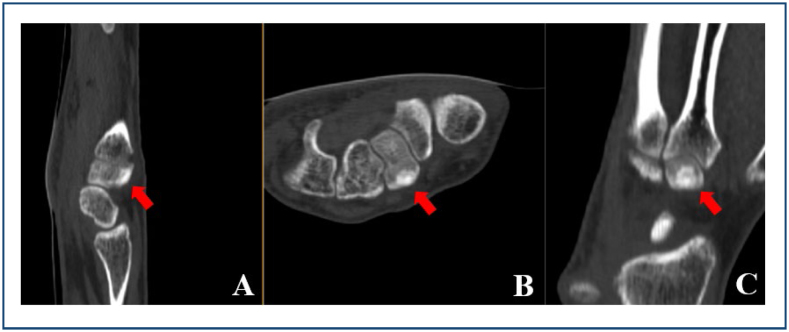
Computed tomography showing a central nidus with surrounding sclerosis in the trapezoid (arrow). **(A)** Sagittal. **(B)** Axial. **(C)** Coronal. Photos cordially provided by Dr. Fábio Augusto Caporrino from Escola Paulista de Medicina, Federal University of São Paulo.

The clinical picture is characterized mainly by nocturnal pain, which responds well to the use of acetylsalicylic acid and non-steroidal anti-inflammatory drugs. Edema, mobility restrictions, and nail deformities are also described. CT (Figure 6) is the exam with the highest sensitivity (93.1%), showing a central nidus with surrounding sclerosis, and is followed by magnetic resonance imaging (MRI) (81.6%). Plain radiography and scintigraphy are also of great value^27^.

The surgical treatments described are en bloc resection or nidus open curettage, followed by bone grafting of the distal radius or iliac bone. Alternative treatments of CT-guided percutaneous ablation of the nidus using radiofrequency (RF), laser photocoagulation (LPC), and thermocoagulation (TCG) are described. In the latter, the main complications described are local recurrence, mainly in TCG, and osteonecrosis in RF^27^.

### Osteoblastoma

Osteoblastoma (OB) is a benign and aggressive primary bone tumor rarely found in the hand bones. It has a higher incidence in patients between 10 and 30 years old, with a predominance in males (3:1). Clinically, progression can vary from slow to rapid and aggressive, with exuberant symptoms such as pain, swelling, and local heat. Unlike OO, generally, it does not respond to the use of nonsteroidal anti-inflammatory drugs (NSAIDs) and the pain does not worsen at night. Plain radiography can show irregularly shaped radio-transparent lesions surrounded by a thin shell of bone. However, these characteristics are not exclusive, being found in other infectious bone pathologies as well as benign and malignant neoplasms. OB is more frequently observed in the cortical bone and less in the innermost part and the juxtacortical regions. Histologically, it is considered benign, even when the lesion appears to be aggressive radiographically, and is similar to OO, although larger. Some authors consider lesions smaller than 1.5 cm as OO and larger than this arbitrary value as OB^28^. Regarding treatment, excision of the tumor, through intralesional curettage or en bloc resection, is the treatment of choice. Chemotherapy and radiotherapy, used in inoperable cases, have demonstrated high levels of recurrence. A recent initial study showed promising results with the use of denosumab in the clinical treatment of first metacarpal OB^29^.

## MALIGNANT BONE TUMORS

### Osteosarcoma

Osteosarcoma is rare in the hand, representing between 0.18 and 0.9% of all osteosarcomas (0.3% of bone sarcomas) and having less than 50 cases reported in the literature^25,30^. Most of them are neoplasms secondary to radiotherapy, Paget's disease, or osteosarcoma metastasis from other regions^31^. In a retrospective series of 402 cases of hand tumors, only one case of osteosarcoma was observed^32^. The 10-year survival rate for osteosarcoma is 63% when located in the hand and wrist regions^30^.

Surgical resection is recognized as an effective basic treatment and, recently, limb-salvage techniques have become standard for patients with osteosarcoma of the limbs, with success rates of 60–80% regions^33^. Classical treatments for osteosarcoma include neoadjuvant chemotherapy, limb-salvage surgery, and adjuvant chemotherapy. The purpose of neoadjuvant chemotherapy is to reduce the tumor and the reactive inflammatory edema to facilitate subsequent resection surgery, as well as to control the primary lesion and eliminate micrometastases early. When well applied, limb-salvage surgery tends to result in better functional scores and greater 5-year survival when compared with amputation^34^.

### Ewing sarcoma

Location in the hand is rare with few cases described in the literature, with the long bones being more affected than the carpal bones. The metacarpal and proximal phalanges are the most involved bones in the hand, with the thumb and middle finger being the most affected^35^.

Typically, it manifests with insidious pain and edema. Radiography generally shows less bone destruction, often in the metaphysodiaphyseal region of a long bone, with ill-defined margins, and many times with a moth-eaten appearance associated with an onion skin periosteal reaction. Magnetic resonance imaging is requested to assess the extent of the disease, generally showing significant involvement of soft tissues.

The classic treatment of Ewing sarcoma is based on chemotherapy and local control, either surgically or with radiotherapy. In recent decades, the advancement of chemotherapy has greatly improved survival, although in cases where metastases are already present, it is not very effective. The use of chemotherapy and radiotherapy can also be useful to shrink the tumor preoperatively, improving the resection margin and often allowing the minimization of local sequelae.

Ewing sarcoma of the hand has a better prognosis in more proximal locations, probably because the primary tumor is also considerably smaller^36,37^.

### Chondrosarcoma

Primary chondrosarcoma is very rare in the hand, being more frequent in the secondary form. Classically, we divide it into primary chondrosarcoma when it originates from previous bone tissue without lesions, and secondary when it originates from an initially benign bone tumor, such as enchondroma or osteochondroma^38^, especially in patients with multiple lesions such as Ollier's disease, Mafucci syndrome, or hereditary multiple osteochondromatosis^39^.

These are tumors that do not respond well to chemotherapy and radiotherapy, with surgical resection being the treatment of choice. Depending on the histological analysis, they are classified as low, medium, or high grades. For high-medium and high-grade lesions, the recommended treatment is wide surgical resection. In low-grade lesions, some authors recommend intralesional curettage with clearly less local sequelae compared with wide resection^40^. They justify the procedure arguing that the tumor rarely metastasizes, although it often presents with intense local aggressiveness.
